# Spirituality and Narrative in Psychiatric Practice: Stories of Mind and Soul

**DOI:** 10.1192/pb.bp.116.054460

**Published:** 2017-04

**Authors:** Paramabandhu Groves

Telling stories is probably as old as human culture. Our ancestors used storytelling to entertain, instruct and make sense of their experience. A psychiatric history, when well taken, should be more than a fact-finding mission to provide a diagnosis and treatment plan. To be effective in providing treatment, helping with healing and promoting recovery, we need to know what matters to our patients. This includes the realm of belief and practice encompassed by the broad term spirituality. A book then that explores both spirituality and narrative is welcome.

*Spirituality and Narrative in Psychiatric Practice*, like the term spirituality, is broad in its scope. On the one hand, we have agnostic atheist Jeremy Holmes describing in his chapter “Meaning without ‘believing’ ” the spiritual nature of mentalising. As he puts it, ‘an intensely practical and loving pathway to spiritual aliveness’. On the other hand, there are writers from a theistic background, such as mental health chaplain Beaumont Stevenson, who considers how God or a higher power may manifest in the everyday stories of patients, providing a greater frame of reference than the story of self that often limits a human's potential. The early chapters give a range of perspectives on narrative. With characteristic clarity Andrew Sims indicates how through careful psychopathological appraisal from attending to the patient's story, it is possible to distinguish between spiritual experiences and psychiatric symptoms. Later chapters explore narrative and spirituality in a wide variety of themes such as affective disorders, offending behaviour, psychosis and the end of life.

The subtitle of the book is *Stories of Mind and Soul* and it is the stories that really shine. To preserve anonymity some are composite – made up from several people's histories or typical examples – and therefore feel somewhat artificial; nevertheless, they engage the reader and serve didactic purposes well. Others are the words of individuals who have been willing to share their stories, and these have a greater ring of authenticity. In particular, the chapter by Jo Barber stands out as an honest and moving account of someone who has struggled with mental health problems and for whom spirituality has been important – at times problematic but often a resource that has supported her ongoing journey of recovery.

As the editors note in their concluding chapter, pressures on service delivery may get in the way of the time and space to listen well to patients' narratives. However, for good psychiatric practice, not taking a good history is a short-cut we can ill afford. This work is a timely reminder of the importance of the fundamental tool of psychiatry and a welcome enjoinder to attend to what is significant to our patients.

